# The Metabolic Syndrome among Postmenopausal Women in Rural Canton: Prevalence, Associated Factors, and the Optimal Obesity and Atherogenic Indices

**DOI:** 10.1371/journal.pone.0074121

**Published:** 2013-09-09

**Authors:** Huiying Liang, Xi Chen, Qiaozhu Chen, Yulin Wang, Xueji Wu, Yaohui Li, Bingying Pan, Huazhang Liu, Ming Li

**Affiliations:** 1 School of Biotechnology, Southern Medical University, Guangzhou, Guangdong Province, People’s Republic of China; 2 Department of Primary Public Health, Canton Center for Disease Control and Prevention, Canton, Guangdong Province, People’s Republic of China; 3 Obstetrics Outpatient Clinic, Guangzhou Women and Children Medical Center, Guangzhou, Guangdong Province, People’s Republic of China; 4 Da An Gene Co., Ltd. of Sun Yat-sen University, Guangzhou, Guangdong Province, People’s Republic of China; University of Padua, Italy

## Abstract

**Objectives:**

This research aimed to (i) determine the prevalence of metabolic syndrome (MetS) and its components; (ii) assess factors associated with MetS, and (iii) define optimal ethnic-specific cutoffs of obesity- and atherogenic-based markers to predict MetS among postmenopausal women in rural Canton.

**Methodology/Principal Findings:**

The Rural Canton Diabetes and Metabolic Disorders Study, a population based cross-sectional study, was conducted during 2011–2012 in Canton. In person interviews, blood glucose and lipid measurements were completed for 4,706 postmenopausal women who did not receive hormone replacement therapy. MetS was diagnosed using criteria of the Joint-Interim-Statement (JIS), the International-Diabetes-Federation (IDF) and the Modified-Third-Adult-Treatment-Panel (M-ATPIII). Age-standardized prevalence of MetS was 38.4%, 28.8%, and 37.1% according to JIS, IDF, and M-ATPIII criteria, respectively. Excellent agreement was observed between three definitions (*κ*≥0.79), in particular between JIS and ATPIII (*κ* = 0.98, 95%CI: 0.97–0.98). Factors positively associated with MetS were living in Southern Canton, personal income, current smoking, higher BMI, and family history of cardiovascular disease. However, regular leisure-time physical activity can have protective effects. The optimal cutoff values for waist-circumference (WC), waist-to-hip ratio (WHR), waist-to-height ratio(WHtR), BMI, HDL-cholesterol to total cholesterol ratio (HDL/TC), HDL-cholesterol to LDL-cholesterol ratio (HDL/LDL), and triglyceride to HDL-cholesterol ratio (TG/HDL) that predicted the presence of MetS were 79.5 cm, 0.86, 0.53, 22.47 kg/m^2^, 0.33, 0.68, and 0.88, respectively.

**Conclusions:**

This study highlights the importance of MetS among postmenopausal women in rural Canton. Our findings contribute to help selecting Cantonese-specific markers to predict MetS and support the need to establish educational program for promoting healthy-lifestyles among this population.

## Introduction

Metabolic syndrome (MetS) is a cluster of metabolic abnormalities characterized by abdominal obesity, hyperglycaemia, decreased high density lipoprotein (HDL), increased triglyceride, and high blood pressure, which predispose the individual at higher risk for both cardiovascular disease (CVD) and type 2 diabetes [1]. Previous studies suggested that men tend to have a higher prevalence of MetS than age-matched, premenopausal women [2], [3], [4]. However, after menopause, this relationship no longer exists, and the prevalence is markedly higher among women than men [5], particularly over the age of 60 [6], [7]. Kim et al. [8] found that postmenopausal status was associated with an increased risk of the MetS independent of normal aging in Korean women, which was further replicated by Cho et al. in Chinese subjects [9]. Thus, identification of postmenopausal women at high risk for MetS has important implications for the reduction of CVD burden.

Adjacent to Hong Kong, Macao, Taiwan and Southeast Asia, Canton is the transportation, industrial, financial and trade center of South China. As one of the first Chinese cities adopting economic reform and open policy in 1979, when compared with inland cities, Canton rural areas are more economically developed and urbanized. However, few studies of MetS have focused on rural populations [10], not to mention postmenopausal women [11]. The average age of the natural menopause in Canton women is 48–50 years [12]. Thus, the Rural Canton Diabetes and Metabolic Disorders Study among women aged ≥50 years (RCDMDS) allowed us (i) to report the first prevalence data of MetS, (ii) to examine factors associated with MetS, and (iii) to explore the optimum obesity and atherogenic indices cutoffs to predict the presence of MetS among postmenopausal women in rural Canton.

## Materials and Methods

### Ethics Statement

The data used in present study are from the RCDMDS, a population-based, cross-sectional survey which obtained data from March 2011 to April 2012 in rural Canton. The sampling methods and survey protocols as well as the quality control were similar to those for the China National Diabetes and Metabolic Disorders Study (CNDMDS) [13], [14]. This survey was conducted according to the principles expressed in the Declaration of Helsinki. Ethical clearance was granted by the Institutional Review Board of Canton Center for Disease Control and Prevention (Canton CDC) (IRB No: 2010-09CSS -01-C). Written informed consent was obtained from each participant before data collection.

### Study Participants

**Figure 1:**
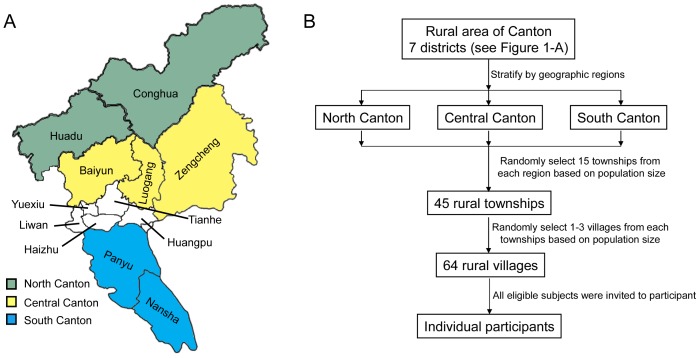
Districts and geographical areas (A) and flowchart (B) of the multistage stratified sampling procedure in the Rural Canton Diabetes and Metabolic Disorders Study (RCDMDS).

In brief, eligible subjects were selected using a multistage stratified, randomized, clustered, sampling technique with probabilities proportionate to size. The total 7 rural districts of Canton were divided into three categories according to geographic region and economic development status as North, Central, and South Canton (Figure.1-A), and 15 rural townships were randomly selected from each part. Within each of the sampled townships, 1–3 rural villages/neighborhoods were randomly selected (Figure.1-B).

All people residing in the villages were officially registered by name, gender and age at the Household Resident Register Record Office of the local police stations (generally do not include migrant workers). Using this register as the sampling frame, all women living in the selected villages and fulfilling the inclusion criteria of an age of ≥50 years were invited to participate in the study. A total of 5,783 individuals were identified and their permanent residency (for more than 5 years) in the village was confirmed through a door-to-door census enrollment. A person was considered ineligible if she had moved out of the village, had not lived there in the past 6 months, or was with other miscellaneous reasons including poor health or hearing problems. Incentives in the form of articles for daily use and free medical examination were given to research participants in order to motivate them to participate in the research.

Of the 5,783 individuals, 5,500 were considered eligible and were enrolled in the study with a response rate of 91.1%. A total of 64 villages and 5,010 participants were finally included in the RCDMDS, representing the rural Canton permanent residential population (≥50 years) of 350,148 in 2010. Reasons for non-participation are refusal (4.2%) and out of area during enrollment (4.7%). For examining the prevalence of the MetS among postmenopausal women, 304 participants were excluded for the following reasons: missing values of the risk factors necessary for a diagnosis of MetS (n = 79); missing data on a questionnaire regarding menopause (n = 26); menopause duration of <1 year (n = 107); premature menopause induced by surgery, chemotherapy, or radiation, and hormone replacement therapy for any cause (n = 92). In the end, 4,706 (93.9%) postmenopausal women were considered eligible for present analysis.

### Data Collection

Examinations were carried out in examination centers at local health stations in the participant’s residential area. A face-to-face interview was conducted by physicians or public health workers from the local township hospital and village health station. All investigators and staff successfully completed a training program that oriented them both to the aims of the study and to the specific tools and methodologies used. Assessed metabolic risk factors included age; anthropometric measurement; personally medical history and family history; and socioeconomic factors including education level, income, and area of residence.

Information on health-related behaviors such as smoking status, alcohol intake, passive smoking and regular physical activity was obtained from the health questionnaire in Cantonese or Mandarin. Current smoking status included smoker and non-smoker. Subjects who currently smoked and had smoked at least 100 cigarettes during her lifetime were classified as current smokers if they answered affirmatively to the following questions: “Do you smoke cigarettes now?” and “Have you smoked at least 100 cigarettes during your lifetime?” [15]. Questions on exposure to passive smoking related to each of the following: childhood (<18 years old), adult home and adult work (yes or no). Individuals who had given up smoking less than a year previously were also classified as smokers. Alcohol consumption was categorized according to the amount and frequency of alcohol consumed: none, current drinker, risk drinker, and high risk drinker [16]. Regular leisure-time physical activity was defined as participation in moderate or vigorous activity for 30 minutes or more per day at least 3 days a week. Socioeconomic status, educational level, occupation, and income were also recorded. A family history of CVD was defined as having at least one parent or sibling diagnosed with diabetes, hypertension, stroke, or coronary heart disease.

Blood pressure, body weight, height, waist circumference (WC), and hip circumference were measured with the use of standard methods, as described previously [13]. BMI was calculated by dividing body weight (kg) by the square of height(m^2^). WC was measured as the smallest circumference between the rib margin and iliac crest, and hip circumference was measured as the maximum circumference between the waist and hip. Waist-to-hip ratio (WHR) and Waist-to-height ratio (WHtR) was calculated by waist circumference divided by hip circumference and height, respectively. Blood pressure was manually measured by trained examiner, 3 times, at 30-s intervals, using the subject’s right arm after a minimum of 10–15 min of rest in a seated position using a mercury sphygmomanometer. The average of the second and third readings was considered as the final blood pressure.

### Blood Glucose and Lipid Measurements

During the survey, blood samples were collected after a 10-h overnight fast, and were properly processed, immediately refrigerated and transported in cold storage to the central laboratory of Canton CDC within 12 h. The fasting time was verified before blood sample collection and participants who had not fasted for at least 10 hours did not have their blood drawn. Plasma glucose was measured by a hexokinase enzymatic method [13], and lipid profile including total cholesterol (TC), HDL, LDL, and triglycerides (TG) levels were determined enzymatically using reagents from the Shino-test Corporation, Japan. Both inter- and intra- assay of variation (coefficient of variation, CV) were less than 3.5% for plasma glucose and serum lipids. Various ratios like TG/HDL, HDL/TC, and HDL/LD were calculated as atherogenic markers.

### Metabolic Syndrome Definitions

MetS was defined based on the most recent 2009 Joint Interim Statement (JIS) criteria [17] (i.e., defined by any three of the following five components: (i) elevated WC [≥80 cm]; (ii) elevated serum TG [≥1.7 mmol/L]; (iii) reduced serum HDL [<1.3 mmol/L]; (iv) elevated blood pressure [systolic blood pressure ≥130 mmHg and/or diastolic blood pressure ≥85 mmHg]; and (v) elevated fasting plasma glucose (FPG) [≥5.6 mmol/L]). For comparison with other reported studies, the International Diabetes Federation (IDF) [18] and modified Third Adult Treatment Panel (ATPIII) [16], [19] criteria were also applied to the dataset.

### Statistical Analysis

All statistical analysis was performed using SPSS (version 16; SPSS, Chicago, IL). Data are presented as means with SE for continuous variables and percentages for categorical variables. Student’s t-test and χ^2^ test were used to determine whether there were differences in the risks for MetS. Non-conditional logistic regression was performed to assess associations between MetS (dependent variable) with familial history, smoking status, passive smoking, alcohol consumption, area of residence and physical activity. The regression model was adjusted for age (continuous), BMI, education, income, marital status, and occupation. Odds ratio (OR) together with its 95%CI and *P* values of the final model are presented. Given the number of missing values are typically small, SPSS will use“pairwise deletion” of the missing values. *P* less than 0.05 was considered significant, and probability values were two-sided.

We calculated the prevalence of metabolic syndrome according to the three criteria and compared their consistence using the Cohen’s Kappa statistic. Cohen’s Kappa coefficient (*κ*) was utilized: the closer *κ* to 1, the better the agreement between the definitions. The age-standardized prevalence rates were calculated with the direct method, using the world population as a standard [20].

Areas under the curve (AUC) for the obesity and atherogenic indices were measured through ROC curve analysis for the diagnosis of MetS among postmenopausal women. The optimal cutoff values of each indice were calculated by plotting the true-positive rate (sensitivity) against the false-positive rate (1-specificity).

To ensure fair and reasonable access to data while preserving the integrity and confidentiality of the information contained in the RCDMDS databases, rigorously “Data Access Guidelines and Procedures” for accessing the databases had been developed in accordance with the China Personal Information Protection and Electronic Documents Act and Canton CDC’s Policy Statement on Privacy and Confidentiality. However, only in exceptional circumstances will Canton CDC release person level data.

## Results

### Prevalence of the Metabolic Syndrome

As presented in Table 1, the crude overall prevalence of MetS defined by the JIS criteria was 37.7%, with a higher age-adjusted estimate (38.4%). In the total study group, the prevalence (crude/adjusted) was lower with both IDF (28.2/28.8%) and modified ATPIII (36.7/37.1%) criteria. Regardless of the definition used, the prevalence of MetS did not change consistently with increasing age under any criteria. Using age-specific rates, peak prevalence was in the 65- to 69-year age-group (JIS criteria: 42.1%; modified ATPIII definition: 41.0%) according to JIS or modified ATPIII criteria, but in the 55- to 59-year age-group (32.8%) according to the IDF definition.

**Table 1:** Prevalence of metabolic syndrome and its individual components among postmenopausal women in rural Canton (n = 4,706).

Age-group (years)	n	Metabolic syndrome			Metabolic syndrome components based on JIS criteria*				
		IDF	JIS	ATPIII	Elevated WC	Elevated TG	Reduced HDL	Elevated BP	Elevated FPG
50–54	568	28.5(162)	39.8(226)	37.3(212)	47.5(270)	44.7(254)	21.8(124)	45.4(258)	43.7(248)
55–59	582	32.8(191)	39.7(231)	38.3(223)	53.6(312)	45.0(262)	21.3(124)	44.7(260)	46.4(240)
60–64	644	25.6(165)	34.6(223)	33.9(218)	48.4(312)	42.2(272)	22.5(145)	45.3(292)	47.5(306)
65–69	912	29.6(270)	42.1(384)	41.0(374)	53.1(484)	46.1(420)	21.6(197)	48.2(440)	48.5(442)
70–74	640	27.8(178)	38.8(248)	38.4(246)	46.2(296)	43.8(280)	22.2(142)	52.8(338)	45.9(294)
75–79	556	28.1(156)	37.1(206)	36.5(203)	46.4(258)	42.4(236)	21.0(117)	54.0(300)	44.6(248)
80–84	468	28.8(135)	36.5(171)	35.3(165)	44.9(210)	34.6(162)	15.4(72)	59.8(280)	44.9(210)
≥85	336	21.1(71)	25.9(87)	25.9(87)	48.2(162)	25.0(84)	13.4(45)	59.5(200)	33.3(112)
Total crude	4706	28.2(1328)	37.7(1776)	36.7(1728)	49.0(23.04)	41.9(1970)	20.5(966)	50.3(2368)	45.3(2130)
Age adjusted	–	28.8	38.4	37.1	49.4	43.3	21.3	47.9	45.6

Data are % (n).

* Elevated WC: waist circumference ≥80 cm, Elevated TG: serum triglycerides ≥1.7 mmol/L, Reduced HDL: serum HDL cholesterol <1.30 mmol/L, Elevated BP: systolic ≥130 mmHg and/or diastolic blood pressure ≥85 mmHg, Elevated FPG: fasting plasma glucose ≥5.6 mmol/L.

### Prevalence of the Individual Components

Table 1 also shows the prevalence of every isolated component according to the JIS definition. The most frequent individual component was high waist circumference (49.4%); the least frequent component was reduced HDL (21.3%). The prevalence of the MetS components did not change consistently with increasing age, with the exception of elevated BP, the prevalence of which increased gradually with increasing age showing a linear association (*P*_trend_<0.001). In subjects with MetS, elevated blood pressure presented the highest prevalence (79.1%), followed by elevated WC (76.6%), elevated FPG (71.5%), elevated TG (67.0%), and reduced HDL (38.1%). As expected, there was a significant difference between postmenopausal women with and without MetS for each of the individual components (all *P* value <0.001, data not shown).

### Metabolic Risk Factors

Presented in Table 2 are selected demographic and risk factor characteristics of MetS for participants in present study. Postmenopausal women with and without MetS were similar with respect to alcohol consumption and exposure to passive smoking. However, subjects with MetS using the JIS criteria were more likely to be married, middle-income (1001–3000 yuan RMB per month), living in South Canton, with lower education level and family history of CVD, without regular leisure-time physical activity, and having a higher BMI.

**Table 2:** Factors associated with metabolic syndrome according to JIS criteria among postmenopausal women in rural Canton (n = 4,706).

	Metabolic syndrome			Unadjusted	Adjusted*
Variables	Non-cases	Cases	*P* value	OR (95%CI)	OR (95%CI)
N	2930(62.3)	1776(37.7)	–	–	–
Residential Area					
North Canton	1123(64.7)	613(35.3)	0.001	1.00	1.00
Central Canton	921(63.1)	539(36.9)		1.07(0.93–1.24)	1.09(0.93–1.27)
South Canton	886(58.7)	624(41.3)		1.29(1.12–1.49)	1.32(1.13–1.54)
Income/person/month					
≤1000 yuan RMB	1442(65.4)	764(34.6)	<0.0001	1.00	1.00
1001–3000 yuan RMB	898(56.1)	702(43.9)		1.48(1.29–1.68)	1.45(1.26–1.67)
3001–5000 yuan RMB	345(65.0)	186(35.0)		1.02(0.83–1.24)	0.96(0.77–1.18)
>5000 yuan RMB	245(66.4)	124(33.6)		0.96(0.76–1.21)	0.97(0.76–1.25)
Married marital status	2093(61.2)	1325(38.8)	0.018	1.18(1.03–1.34)	1.02(0.88–1.18)
Primary/above education	1448(60.5)	944(39.5)	0.013	0.86(0.77–0.97)	0.92(0.80–1.06)
Current smoker	59(54.6)	49(45.4)	0.098	1.38(0.94–2.03)	1.65(1.09–2.51)
Passive smoking					
None	1340(63.9)	756(36.1)	0.664	1.00	1.00
Childhood only	139(60.7))	90(39.3)		1.15(0.87–1.52)	1.14(0.86–1.51)
Adult at home only	115(62.2)	70(37.8)		1.08(0.79–1.47)	1.06(0.78–1.45)
Adult at work only	238(59.8)	160(40.2)		1.19(0.96–1.48)	1.17(0.93–1.45)
Childhood+adult at home	214(61.3)	135(38.7)		1.12(0.89–1.41)	1.11(0.88–1.40)
Childhood+adult at work	232(60.7))	150(39.3)		1.15(0.92–1.43)	1.14(0.91–1.43)
Adult at home+ at work	286(60.6)	186(39.4)		1.15(0.94–1.42)	1.16(0.94–1.42)
Childhood+adult at home+at work	366(61.5)	229(38.5)		1.11(0.92–1.34)	1.09(0.90–1.32)
Alcohol					
None	2807(62.3)	1701(37.7)	0.579	1.10(0.77–1.57)	1.13(0.77–1.65)
Current drinker	89(64.5)	49(35.5)		1.00	1.00
Risk/above drinker	34(56.7)	26(43.3)		1.39(0.75–2.58)	1.17(0.61–2.26)
Exercise (times/week)					
No	2195(59.0)	1524(41.0)	<0.0001	1.00	1.00
1–2	244(68.3)	113(31.7)		0.67(0.53–0.84)	0.72(0.56–0.92)
≥3	491(77.9)	139(22.1)		0.41(0.33–0.50)	0.36(0.30–0.49)
BMI (kg/m^2^)					
<18.5	476(86.2)	76(13.8)	<0.0001	0.31(0.24–0.40)	0.30(0.23–0.39)
≥18.5 and <25	1973(65.8)	1025(34.2)		1.00	1.00
≥25 and <30	411(42.1)	565(57.9)		2.65(2.28–3.07)	2.61(2.24–3.04)
>30	70(38.9)	110(61.1)		3.03(2.22–4.12)	2.83(2.06–3.89)
Family history of CVD					
No	2897(62.7)	1721(37.3)	<0.0001	1.00	1.00
Yes	33(37.5)	55(62.5)		2.81(1.82–4.34)	2.24(1.41–3.56)

Data are % (n).

* Multivariable-adjusted odds ratio. Age was inserted as a continuous variable.

Multivariable analysis revealed that the significant independent variables (risk factors) for MetS in the final model included living in South Canton (OR 1.32 [95% CI 1.13–1.54]), with middle income (OR 1.45 [95% CI 1.26–1.67]), being current smoker (OR 1.65 [95% CI 1.09–2.51]), higher BMI (OR 2.61 for 25≤BMI<30 kg/m^2^ and 2.83 for BMI≥30 kg/m^2^ [95% CI 2.24–3.04 and 2.06–3.89, respectively]), and family history of CVD (OR 2.24 [95% CI 1.41–3.56]); lower BMI (<18.5 kg/m^2^) and regular leisure-time physical activity were protective factors (Table 2). Level of education and marital status, although significant in univariate analysis, failed to achieve significance in multiple logistic regression.

### Optimization of Obesity and Atherogenic Indices

**Figure 2:**
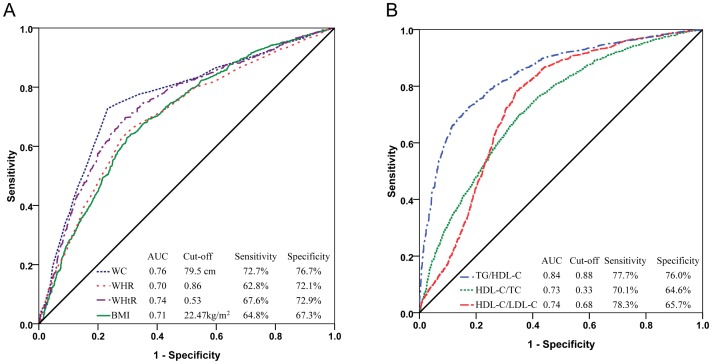
The ROC (receiver operating characteristic) curves for obesity (A) and atherogenic (B) indices to predict the presence of metabolic syndrome (MetS), as defined by the 2009 JIS criteria, among postmenopausal women in rural Canton.

The AUCs of those obesity and lipid-based markers which showed significant predication of MetS among postmenopausal women is shown in Figure 2. The optimal cut points to detect MetS in postmenopausal women were 79.5 cm (for WC), 0.86 (for WHR), 0.53 (for WHtR), 22.47 kg/m^2^ (for BMI), 0.88 (for TG/HDL), 0.33 (for HDL/TC), 0.68 (for HDL/LDL) and the corresponding sensitivity and specificity were 72.7% and 76.7%, 62.8% and 72.1%, 67.6% and 72.9%, 64.8% and 67.3%, 77.7% and 76.0%, 70.1% and 64.6%, 78.3% and 65.7%, respectively.

### Comparison and Agreement between the JIS and IDF or ATPIII Definitions

Table 3 reports the agreement between the 3 definitions according to age groups. Among all participants, 98.98% were classified similarly under the JIS and modified ATPIII definitions (*κ* = 0.98 [95% CI 0.97–0.98]), fair agreement when comparing modified ATPIII and IDF definitions (91.50%, *κ* = 0.81 [95% CI 0.79–0.83]). Lower agreement (90.48%, *κ* = 0.79 [95% CI 0.77–0.81]) was found between the JIS and IDF definitions. However, all of the positive subjects (1,728 and 1,328), who were diagnosed by modified ATPIII and IDF definition, respectively, were also identified as positive following of JIS definition. Despite the general excellent concordance for the entire population, differences were noted between age groups. Agreement between the different age-specific prevalence rate estimates ranged from 0.73 to 1.00.

**Table 3:** Agreements* between the JIS, ATPIII-modified, and IDF criteria in diagnosing metabolic syndrome among postmenopausal women in rural Canton (n = 4,706).

	JIS *vs* ATPIII	JIS *vs* IDF	ATPIII *vs* IDF
Total	98.98±0.15 (0.98)	90.48±0.43 (0.79)	91.50±0.41 (0.81)
Age group (years)			
50–54	97.54±0.65 (0.95)	88.73±1.33 (0.75)	91.20±1.19 (0.80)
55–59	98.63±0.48 (0.97)	93.13±1.05 (0.85)	94.50±0.95 (0.88)
60–64	99.22±0.35 (0.98)	90.99±1.13 (0.79)	91.77±1.08 (0.81)
65–69	98.90±0.35 (0.98)	87.50±1.10 (0.73)	88.60±1.05 (0.75)
70–74	99.69±0.22 (0.99)	89.06±1.24 (0.76)	89.38±1.22 (0.76)
75–79	99.46±0.31 (0.99)	91.01±1.21 (0.80)	91.55±1.18 (0.81)
80–84	98.72±0.52 (0.97)	92.31±1.23 (0.83)	93.59±1.13 (0.85
≥85	100.00±0.00 (1.00)	95.24±1.16 (0.87)	95.24±1.16 (0.87)
*P*-value	0.004	<0.0001	<0.0001

Data are Percent±SE (*κ*).

* Percentage of participants who were classified as either having or not having the metabolic syndrome under the considered definitions of the metabolic syndrome.

## Discussion

This study provides new population-based data on the prevalence of MetS in Cantonese postmenopausal women aged ≥50 years living in rural village setting. In this current study, 38.4% of the women met JIS criteria, 28.8% and 37.1% of the women met current IDF and ATPIII-modified criteria. To the best of our knowledge, this is the first large scale, population-based, cross-sectional survey to estimate the prevalence of MetS in Chinese postmenopausal women. Available data on the prevalence of MetS from epidemiology studies among Chinese postmenopausal women is limited to those using IDF criteria, with small sample sizes, and only crude prevalence rates reported [7], [21]. Based on the IDF criteria, a study of 225 postmenopausal women older than 60 years in Chengdu, China, showed that the crude prevalence of MetS was 37.3%, which was higher than that in our study (28.2%), possibly because their participants were older [7]. Another study of 181 postmenopausal women in Beijing, China, showed that the prevalence of MetS was 33.7%, which was also higher than that in our study, possibly because their participants were recruited through advertisements in newspapers [21]. Those recruited through advertisement contain a selection bias for participants at the stage of seeking out help or treatment.

According to the JIS criteria, the most frequent individual component was high waist circumference in postmenopausal women and high blood pressure in subjects with MetS. Some studies have reported a strong association between menopause and increased central adiposity [22] and blood pressure [23]. Both high waist circumference [24] and high blood pressure are known risk factors for CVD [25]; these findings may suggest that if the current trend continues, this population is at increased risk for CVD. Traditionally, mortality from coronary heart disease in China has been infrequent and is estimated to be only 10% of that in Western populations [26]. In contrast, cerebrovascular disease (eg. stroke) is more common in Chinese population, especially in rural areas [27]. The least common individual component was low HDL in both total postmenopausal women and subjects with MetS; similar findings have been reported in Beijing [21] and Chengdu [7], China. Whether this contributes to the low risk of coronary artery disease in Chinese population is unclear and would require further studies to establish its significance.

Multiple Logistic regression analyses were conducted to examine the association of independent variable (literacy, marital status, income, residential area, smoking, exposure to passive smoking, alcohol consumption, physical activity, BMI and family history of CVD) with MetS. Factors positively associated with MetS in our study were living in Southern Canton, with middle income, being current smoker, higher BMI and family history of CVD. In contrast, regular leisure-time physical activity and lower BMI were negatively associated with MetS. Contrary to some [28], [29], but in line with others [30], we did not find any association between passive smoking and MetS, neither between the alcohol consumption and MetS. These data suggest that to combat the MetS epidemic among postmenopausal women in Rural Canton, interventions on diet and lifestyle modification will be an effective and efficient approach in the prevention and control of the syndrome. However, sedentary lifestyle was becoming prevalent, especially in Southern Canton. Unfortunately, such a trend is still going on.

Comparisons of the prevalence of MetS among different populations are generally difficult due to its varying definitions specified by different international authority. By comparing the three last definitions, the prevalence of JIS MetS was higher than IDF and modified ATPIII MetS. Concerning the agreement between definitions, although the IDF placed more emphasis on central obesity in the causation of the MetS, good levels of agreement were found with the JIS (*κ* = 0.79) and modified ATPIII (*κ* = 0.81), indicating that the requirement of abdominal obesity did not induce important discrepancies in the prevalence or the classification of the MetS. Similar results have been observed in a previous large Chinese population [31] and other Caucasian populations [32], [33].

Wildman *et al.* [34] found that the rate of obesity among Chinese people, particularly in Southern China, is lower than that among people in Western countries. To our knowledge, this is the first report in Southern China that attempts to define obesity and atherogenic indices cut points to predict MetS among postmenopausal women. The cut-off values of the markers to detect the MetS in Rural Canton postmenopausal women are 79.5 cm, 0.86, 0.53, 22.47 kg/m^2^, 0.88, 0.33, and 0.68 for WC, WHR, WHtR, BMI, TG/HDL, HDL/TC, and HDL/LDL, respectively. This finding partially agrees with a similar study conducted among Ghanaian postmenopausal women by Arthur *et al.* [35], which identified cut-off for WC, WHR, and HDL/TC to be 80.5 cm, 0.84, and 0.34, respectively. However, WC and BMI cut-off points are lower than that reported in Northern Chinese postmenopausal women (80.75 cm and 24.84 kg/m^2^) [21]. In general women in China are defined as being overweight with BMI of 24 kg/m^2^ but with a cut-off point of 22.47 kg/m^2^ identified in study groups, there is the possibility that Cantonese women develop MetS at a lower anthropometric indices than the Northern Chinese populations.

Strengths of present study are (i) it is based on recent nationwide, population-based, representative sample of postmenopausal women aged ≥50 years, who were ignored by most studies; (ii) all variables were measured using standard methods and vigorous quality control; and (iii) seven districts involved in present study were typical rural areas nearby modern region–Canton, one of the most modernized cities in China, experiencing rapid adaptation to a westernized lifestyle (low physical activity, high fat/low fiber diets). Nevertheless, several limitations deserve mentioning.

First, the cross-sectional design of the RCDMDS study limits the possibility to determine which criteria better predicts adverse cardiovascular outcomes, such as the incidence of cerebrovascular events and thus precludes causal inferences. Second, the history of heart disease, hypertension, diabetes, and family history of CVD in this rural population might be inaccurate and might not fully identify those with health problems because health care in rural areas is not routinely obtained. Third, selection bias might have occurred during the exclusion of ineligible women, particularly the postmenopausal women under the age of 50. Consequently, subjects included in our analysis were elder and had a lower education level. These differences indicate that the nonparticipants likely had lower rates of MetS; therefore, our estimates may be higher than the true postmenopausal women prevalence. Finally, some factors, which may influence the observed associations, could not be considered. For example, in China, 52.9% of men are reported to be current smokers, compared with only 2.4% of women [36]; thus, passive smoking will be important to women. However, the number of years of exposure to passive smoking was not considered in this investigation. Other factors that could not be considered in the present study included information on number of pregnancies, hypertension in pregnancy, pre-eclampsia, gestational diabetes, and so on. Further studies are warranted that include broader risk factors known to be related to MetS progression of women.

## Conclusions

In conclusion, our study indicated that MetS was highly prevalent among postmenopausal women in Canton rural areas regardless of which criterion used to define MetS. Elevated WC and elevated BP are the most frequent characteristics in comparison to other metabolic components. Living in South Canton, personal income, current smoking, higher level of BMI, and family history of CVD increased the MetS risk, whereas regular leisure-time physical activity could have protective effect on MetS. This study also found sensitive Cantonese specific obesity and atherogenic markers to identify the presence of MetS among postmenopausal women.

Thus, one of the principle implications of our findings underscores the importance of promoting healthy lifestyles, such as weight management, adequate physical activity, and quit smoking for the prevention of MetS in rural Canton and possibly in other developing countries. Another implication is that our findings will provide healthcare providers a useful tool to identify postmenopausal women who are at great risk of developing MetS and focus more efforts on this special population.
